# Combined occurrence of diabetes mellitus and retinitis pigmentosa

**DOI:** 10.4103/0256-4947.59381

**Published:** 2010

**Authors:** Afaf Al-Adsani, Fadl Abdel Gader

**Affiliations:** From the Department of Medicine, Al-Sabah Hospital, Kuwait

## Abstract

The combined occurrence of diabetes mellitus (DM) and retinitis pigmentosa (RP) is rare. It has been reported in the form of four different syndromes that are inherited in an autosomal recessive fashion. We describe two cases of DM and RP occurring together. The first case was a 35-year-old male who presented with insulin-treated diabetes, obesity, hypertension, polydactyly, normal cognitive functions, an ataxic gait, blindness secondary to RP, dyslipidemia, impaired renal function, and multiple renal cysts. He was diagnosed clinically as having Bardet-Biedl syndrome. The second case was a 34-year-old male who presented with insulin-resistant diabetes, hypertension, blindness secondary to RP, deafness, normal cognitive functions, primary infertility, renal, and liver impairment. He was diagnosed clinically as having Alström syndrome. Because of overlapping clinical manifestations and the cost and time involved in genetic studies, clinical criteria can be used for diagnosis and as a guide for genetic mapping in these patients.

Although diabetes mellitus (DM) and retinitis pigmentosa (RP) are common and occur independently, the combination of both conditions is rarely seen in clinical practice. Four syndromes have been reported^2^,^8^,^15^,^20^ describing the combination of both conditions that were coexistent with other manifestations such as obesity, deafness, hypogonadism, renal abnormalities, and/or mental retardation. The inheritance of these syndromes is autosomal recessive. Diagnosis can be done clinically based on primary and secondary features, but molecular genetic testing may be needed for accurate diagnosis and genetic counselling. In this report, we describe two cases who presented with diabetes and retinitis pigmentosa along with other manifestations. In both cases, the diagnosis was established on the basis of clinical findings. The first case was diagnosed as Bardet-Biedl syndrome based on the clinical presentation of DM with RP along with obesity, polydactyly, renal cortical cysts, and ataxic gait, whereas the second case was diagnosed as Alström syndrome based on the clinical presentation of RP and insulin-resistant diabetes, deafness, hepatic dysfunction, renal impairment, and hypogonadism. We also reviewed the literature summarizing the clinical manifestations and the mode of inheritance of each syndrome.

## CASE 1

**Figure 1:**
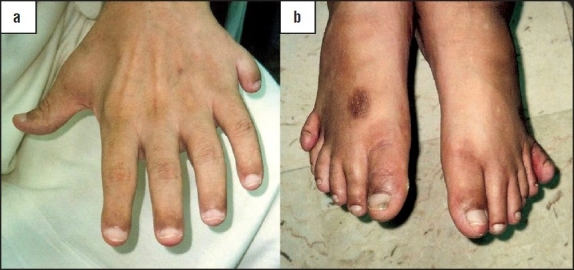
Polydactyly of left hand (a) and both feet (b).

**Figure 2:**
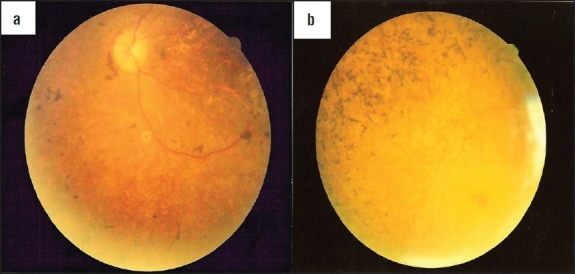
Retinitis pigmentosa: a speckling of the retinal pigment epithelium with bone-spicule pigmentation, optic nerve pallor, and retinal arteriolar attenuation.

A 35 year-old Iraqi male was referred to our diabetes clinic for insulin-treated DM, impaired renal function with a serum creatinine level of 188 mmol/L (normal reference range is 45-105 mmol/L), gross proteinuria of 2.7 g/day, and a fasting blood sugar level of 13.7 mmol/L (RR is 3.9-6.1 mmol/L). The patient had been diagnosed with Laurene-Moon-Biedl syndrome since 1990, with a history of night blindness and gradual visual deterioration since childhood. He was ultimately registered as being blind in 1999, when he was also found to be diabetic. He was hypertensive and dyslipidemic; his parents were first cousins. Clinical examination revealed a weight of 120 kg, a height of 180 cm, a body mass index (BMI) of 36.3 kg/m^2^, and a BP of 140/100 mm Hg. He had polydactyly of the left hand and both feet (Figure 1). Examination of the heart, abdomen, and chest did not reveal any abnormalities. Neurologically, he had normal cognitive functions, but had an ataxic gait and diminished vibration and pain sensation over both the lower limbs with intact peripheral pulsation. Fundus examination revealed retinitis pigmentosa and optic atrophy (Figure 2, Panel a). His biochemical profile showed an elevated triglyceride level at 3.54 mmol/L (RR is 0-2.2 mmol/L) and an LDL cholesterol level of 2.28 mmol/L (RR is 3.9-5.2 mmol/L). Serum creatinine level gradually rose to 290 mmol/L; the C-peptide level was 137 pmol/L (RR is 256-1325 pmol/L). Abdominal ultrasound showed multiple cortical renal cysts with multiple stones, mild hepatomegaly, and multiple gall bladder stones. Molecular genetic testing was not available. His HbA_1c_ level of 6.2% was controlled with insulin. He was diagnosed clinically as having Bardet-Biedl syndrome based on the clinical presentation of DM with retinitis pigmentosa, obesity, polydactyly, renal cortical cysts, and an ataxic gait.

## CASE 2

34-year-old Kuwaiti male was referred to our diabetes clinic for insulin-treated diabetes and uncontrolled hyperglycemia. A detailed history revealed that diabetes was diagnosed five years ago along with a diagnosis of hypertension. He begin having gradual visual deterioration and night blindness at the age of six years and was registered as being blind at the age of nine years. His parents were third cousins and his sister, who was also diabetic and blind, had died of renal failure. He had been married for one year; primary infertility was also observed. Clinical examination revealed a weight of 71 kg, a height of 158 cm, a BMI of 28.4 kg/m^2^, and a blood pressure of 120/80 mm Hg. Fundus examination revealed retinitis pigmentosa along with optic atrophy (Figure 2, Panel b). Systemic examination did not reveal any remarkable findings, instead showing intact peripheral sensation and pulsation. Investigations revealed a fasting blood glucose level of 18.1 mmol/L, HbA_1c_ of 15.1%, and a C-peptide level of 2247 pmol/L (normal range: 256-1325 pmol/L). The triglyceride level was 4.06 mmol/L, LDL cholesterol level was 4.25 mmol/L, and HDL cholesterol level was 1.07 mmol/L (RR is 1.01-2.94 mmol/L). His serum creatinine level was 96 μmol/L and microalbuminurea testing yielded 457 mg/day (RR is < 20 mg/day). The alkaline phosphatase level was 155 U/L ((RR is 50-136 U/L)) and ALT 63 U/L (RR is 30-65 U/L). Thyroid function tests were normal. The audiogram demonstrated sensineural deafness. Endocrinological evaluation revealed azospermia and biopsy showed only Sertoli cells. Genetic testing yielded 46XY, but no chromosomal mutations, deletions or other abnormalities could be detected. Molecular genetic testing was not available. His diabetes was gradually controlled with increasing doses of insulin and metformin given at doses of up to 2 g per day along with statins. This resulted in better glycemic control and significant improvement in the lipid profile and HbA_1c_ level of 9.7%. Subsequently, metformin was discontinued because of elevated liver enzyme levels. Abdominal ultrasound revealed a fatty liver and liver biopsy revealed nonalcoholic steatohepatitis. Screening for hepatitis viruses B and C as well as for ANCA and ANA antibodies was negative. Following improvement of the liver profile, rosiglitazone was prescribed along with insulin, and HbA_1c_ improved to 8.8%. Later, the renal profile was found to be impaired and ultrasound examination of the liver showed cirrhotic changes. The serum creatinine level remained stable at 120 μmol/L, and he declined renal biopsy. Rosiglitazone was discontinued and the insulin dose increased up to 245 units per day. His blood sugar levels were still out of control and he was not adherent to his controlled diet regimen. He was diagnosed clinically as having Alström syndrome based on his clinical presentation of insulin-resistant diabetes with retinitis pigmentosa, deafness, hepatic dysfunction, renal impairment, and hypogonadism.

## DISCUSSION

RP is a group of inherited disorders characterized by progressive peripheral vision loss and night vision difficulties that can lead to central vision loss. It is caused by molecular defects in more than 100 different genes.^1^ RP is most commonly found in isolation, but it can be associated with several systemic diseases such as deafness, neurological disorders, cardiovascular anomalies, renal manifestations, obesity, and diabetes. Four rare syndromes have been reported as describing the combination of RP and DM. These include Bardet-Biedl syndrome, Alström syndrome, Kearns-Sayre syndrome, and rarely, Wolfram syndrome. Table 1 summarizes the mode of inheritance, the affected genes, and the clinical manifestations of each syndrome.

**Table 1:** Syndromes associated with diabetes mellitus and retinitis pigmentosa

	Bardet-Biedl	Alström	Kearns-Sayre	Wolfram (DIDMOAD)
Mode of inheritance	Autosomal recessive	Autosomal recessive	Mitochondrial disease	Autosomal recessive
Genes involved	12 genes, mostly BBS1, BBS2, BBS6, BBS10, MKKS	ALMS1 gene, 81 mutations so far	mt-DNA mutations/deletions	WFS1 gene mutation
Clinical features				
Diabetes mellitus	Noninsulin-dependent	Insulin-resistant	Insulin-dependent	Type 1 nonautoimmune
Retinitis pigmentosa	+	+	+	+
Childhood obesity	+	+	−	−
Mental retardation	+	−	−	−
Polydactyly	+	−	−	−
Infertility/hypogonadism	+	+	+	−
Sensorineural deafness	−	+	−	+
Renal disease	+	+	−	−
Hepatic involvement	+	+	−	−
Short stature	±	+	+	−
Acanthosis nigricans	−	+	−	−
Cardiac disease	Congenital detects	Cardiomyopathy	Heart block	−
Neurologic disease	Ataxia, incoordination	Absence seizures, sleep disorders	External ophthalmoplegia	Ataxia, brain stem disorder, nystagmus, sleep apnea
Other features	−	Hypothyroidism, hypertension	Hypothyroidism Hypomagnesemia, Hyperaldosteronelism	−
Prognosis	Death is usually due to renal failure and 25% die by the age of 44 years	Death is usually due to renal failure and patients rarely live longer than 40 years.	Death is usually from cardiac disorders and death is common in the third or fourth decade of life	Death is usually due to respiratory and renal failure and median age at death is 30 years

The term, “Laurence-Moon-Bardet-Biedl” was coined in 1925.^2^ In 1982, Laurence-Moon and Bardet-Biedl syndromes began to be considered as two separate syndromes due to the essential differences between them.^3^ The Laurence-Moon syndrome is considered to be composed of retinitis pigmentosa, obesity, hypogonadism and spastic paraparesis without polydactyly, and DM. However, recently, and because mutations have now been detected in Bardet-Biedl syndrome genes in families conforming to the diagnosis of the Laurence-Moon syndrome, some authorities do not believe in this distinction and consider the two to be one syndrome under the label of Bardet-Biedl syndrome (BBS). The prevalence of BBS ranges from one in 13 500 in a consanguineous population, to one in 160 000 in nonconsanguineous populations. It is usually inherited in an autosomal recessive manner.^2^ It is clinically characterized by: (i) cone-rod dystrophy (100%) that usually affects the vision in the first decade; the patients will be legally blind by the second decade, (ii) obesity (72-96%), which usually begins within the first year, (iii) postaxial polydactyly (58-69%), (iv) renal dysfunction (46%), (v) cognitive impairment, (vi) male hypogonadotrophyic hypogonadism, and (vii) complex female genitourinary malformations. All above manifestations are postulated to be due to defects in the cilia. Because of the wide range of clinical variability observed within BBS, the diagnosis is established on the basis of clinical findings. Clinical diagnosis can be made with four primary or three primary and two secondary features as shown Table 2.^2^ Diabetes mellitus is diagnosed in 32-45% of cases with BBS.^3^,^4^ It is usually noninsulin-dependent diabetes but is occasionally insulin-dependent, and is treated as usual in the general population.^2^,^4^ Renal disease is a major cause of morbidity and mortality in BBS patients and 25% die by the age of 44 years.^4^ Molecular genetic studies found 14 genes to be associated with BBS.^2^,^5^,^6^,^7^ Table 3 summarizes the genes identified so far and the percentage of the most common causative mutations and exons. However, approximately 20% to 30% of patients do not have identifiable mutations, suggesting that more BBS cases are yet to be identified. Some genotype/phenotype correlations have been reported (Table 3) although most of the studies did not confirm these correlations.

**Table 2:** Diagnostic criteria for Alström syndrome and Bardet-Biedl syndrome.

	Major criteria/Primary features	Minor criteria/Secondary features	Other supportive evidence	Diagnosis
Bardet-Biedl syndrome	Cone-rod dystrophy	Speech disorder/delay		
	Postaxial polydactyly	Strabismus/cataracts/astigmatism		
	Truncal obesity	Brachydactyly/syndactyly		
	Learning disabilities	Developmental delay		
	Hypogonadism in males or genital abnormalities in females	Polyuria/polydipsia (nephrogenic diabetes insipidus)		
	Renal anomalies	Ataxia/poor coordination/imbalance		4 major criteria OR 3 major + 2 minor criteria
		Mild hypertonia (especially lower limbs)		
		Diabetes mellitus		
		Dental crowding/hypodontia/small dental roots/high-arched palate		
		Cardiovascular anomalies		
		Hepatic involvement		

Alström syndrome	ALMS 1 mutation in 1 allele and/or family history of Alström syndrome	Obesity and/or insulin resistance and/or type 2 diabetes	Recurrent pulmonary infections	
	Vision (legal blindness, history of nystagmus in infancy/childhood, cone and rod dystrophy by electroretinogram	History of dilated cardiomyopathy/congestive heart failure	Normal digits	
		Hearing loss	History of developmental delay	2 major + 2 minor criteria
		Hepatic dysfunction	Hyperlipidemia	
		Renal failure	Scoliosis	
		Short stature	Flat wide feet	OR
		Males – hypogonadism	Hypothyroidism	
		Females – irregular menses and/or hyperandrogenism	Hypertension	1 major + 4 minor criteria
			Growth hormone deficiency Alopecia	
			Recurrent urinary tract infections or urinary dysfunction	

**Table 3:** Known genes and gene mutations in Bardet-Biedl syndrome and Alström syndrome.

	Causative genes identified	Mutations (%)	Exon (%)	Phenotype correlations
Bardet- Biedl syndrome	14 genes^5^			
	BBS1^2^	- 11q13 (23.2%)^2^	- 12 (46.7%), 16 (16.7%), 15 (6.7%), 11 (6.7%), 4 (10%), 13 (3.3%), 10(3.3%), 8(3.3%), 1(3.3%)^2^	
	BBS2^2^	- 16q21 (8.1%)^2^	- 2 (30%), 1 (15%), 4 (15%), 8 (10%), 6 (10%), 14 (10%), 9 (5%), 15 (5%), 3 (5%)^2^	- BBS2, BBS3, and BBS4 correlated with characteristic ocular phenotypes.
	ARL6/BBS3^2^	- 3p12-q13 (0.4%)^2^		
	BBS4^2^	- 15q22.3-q23^2^	- 13 (16.7%), 12 (16.7%), 8 (16.7%), 4 (16.7%), 3-4 (16.7%), 15 (8.3%), 7 (8.3%)^2^	BBS4 correlated with extra digits.
	BBS5^2^	- 2q31 (0.4%)^2^	- 7	
	MKKS/BBS6^2^	- 20p12 (5.8%)^2^	- 3 (80.7%0, 6 (12.9%), 4 (3.2%), 5 (3.2%)^2^	BBS2, BBS3, BBS4, and
	BBS7^2^	- 4q27 (1.5%)^2^	- 10 (50%), 6 (25%), 7 (25%)^2^	BBS6 correlated with adult obesity.
	TTC8/BBS8^2^	- 14q32.1 (1.2%)^2^	- 10^2^	BBS4 and BBS6 correlated with childhood obesity.
	B1/BBS9^2^	- 7p14 (NK)^2^		
	BBS10^2^	- 12q21.2 (20%)^2^		
	TRIM32/BBS11^2^	- 9q31-q34.1 (<0.4%)^2^		
	BBS12^2^	- 4q27 (5%)^2^		
	MKS/BBS13^2^	- 17q23^6^		
	MKS/BBS14^2^	- 12q21.37		
Alström syndrome	81 mutations^8^	2p1^2^	- 16 (40%)^8^	Exon 16 correlated with more severe disease, early RP, urologic dysfunction, dilated cardiomyopathy, and diabetes.
	ALS1		- 10 (23%)^8^	Axon 8 is correlated with renal disease.
			- 8 (21%)^8^	
			- 6^8^	
			- 12^8^	
			- 17^8^	
			- 18^8^	

ARL6, ADP-ribosylation factor-like 6 gene; MKKS, McKusick-Kaufman syndrome; TTC8, Tetratricopeptide repeat domain 8 gene; TRIM32, Tripartite motif-containing 32 gene; MKS, Meckel-Gruber syndrome^2^,^5^,^6^,^7^,^8^

Alström syndrome is a rare autosomal recessive genetic disease characterized by multi-organ dysfunction. It is clinically characterized by: (i) childhood obesity (95%), (ii) progressive cone-rod dystrophy ultimately leading to blindness with 90% of the patients becoming blind at the age of 16 years, (iii) sensorineural deafness (80%), (iv) dilated cardiomyopathy (60%), (v) heptic dysfunction (80%), (vi) renal insufficiency (50%), and (vii) endocrinological features.^8^ It was first described in 1959 and 500 cases have been detected globally so far without any gender predilection.^8^ It is now considered to be one of the class of genetic diseases known as ciliopathies in which the ciliary function of various cell types are affected, resulting in dysfunction of cells of renal tubules, sperm cells, and retinal cells, thereby producing the components of the syndrome.^9^ The genetic disease is due to mutations in the *ALMS1* genes, of which 81 mutant genes have been detected so far (Table 3).^8^ As in Bardet-Biedl syndrome, and because of the wide-ranging and complex spectrum of phenotypes reported in the literature, Marshall et al adopted the diagnostic criteria for Alström syndrome as shown in Table 2.^10^ In adults, two major criteria and two minor criteria, or one major and four minor criteria are required for the clinical diagnosis of the syndrome. Hyperinsulinemia, which develops in early childhood, has been reported in 92% of individuals with ALM. Type 2 DM is diagnosed in 82% of the affected individuals older than 16 years of age and it is usually diagnosed in the second or third decade.^8^,^11^ Hyperinsulinemia and hyperglycemia may be managed with a low carbohydrate diet, metformin, and rosiglitazone.^12^,^13^ Cognitive function is preserved in Alström syndrome and polydactyly is not a feature, thus distinguishing it from Bardet-Biedl syndrome. Dilated cardiomyopathy can occur at any age, but it mainly occurs in infancy and is the main cause of mortality in that age group, whereas renal failure is the main cause of mortality in elder patients.^14^ The life span of patients with Alström Syndrome rarely exceeds 40 years.^13^

Kearns-Sayre syndrome (KSS) is a rare mitochondrial disease with an as yet undetermined mode of inheritance. The true prevalence of this syndrome is unknown. The mitochondrial abnormalities involve mitochondrial DNA mutations, rearrangements, or deletions. It was first described in 1958 and several hundred cases have been diagnosed to date. Its labile clinical features make it rather difficult to diagnose.^15^ It is characterized by: (i) external ophthalmoplegia, (ii) retinitis pigmentosa, (iii) cardiac conduction defects, (iv) a variety of endocrine abnormalities including short stature, gonadal failure, diabetes mellitus, thyroid disease, hyperaldostronism, hypomagnesemia, and (v) bone and dental abnormalities.^16^ Diabetes mellitus was recorded in 13% of cases with KSS,^16^ and was of the insulin-dependent type due to insulin deficiency.^17^ KSS cases have high mortality rates which is directly attributable to the cardiovascular manifestations of this syndrome.^18^

Wolfram syndrome (WFS) is an autosomal recessive neurodegenerative disorder that predisposes individuals to the development of type 1 diabetes in association with progressive optic atrophy. It is an extremely rare condition with a prevalence of 1 in 770 000.^19^ It is also known as DIDMOAD syndrome, the name being derived from diabetes insipidus, diabetes mellitus, optic atrophy, and deafness. RP was reported in a few cases.^20^ The genetic basis of the disease is due to mutations in the WFS1 gene, which has been postulated to be an endoplasmic reticulum calcium channel transporter in pancreatic β-cells and neurons.^18^ Although diabetes and optic atrophy represent the minimal diagnostic criteria, other clinical characteristics include neurodegenerative disease involving the hypothalamus, brain stem, and the cerebellum. Diabetes is usually diagnosed in childhood and is of the nonautoimmune insulin-dependent type.^21^ Deafness is not always a prominent component. Other abnormalities include dilated renal outflow tracts and primary gonadal atrophy. Death is usually due to respiratory failure from brain stem involvement^22^ and renal failure.^19^

Of note, patients with DM and RP do not develop signs of diabetic retinopathy. The absence of diabetic retinopathy in RP was explained by the suggestion that the loss of photoreceptors reduced glycolysis and decreased the production of free radicals.^23^ The combination of diabetes and blindness in these syndromes confers a high risk of foot ulceration. However, it has been reported that patients with Alström syndrome are protected from peripheral sensory neuropathy.^24^

In conclusion, we report two cases who had presented with retinitis pigmentosa and diabetes mellitus along with other overlapping clinical manifestations. Both cases were diagnosed based on clinical features into two different pleiotropically genetic disorders, namely, the Bardet-Biedl syndrome and Alström syndrome. Due to the overlapping clinical manifestations in these genetic disorders and the cost and time usually needed for genetic studies, clinical criteria can be used for diagnosis and as a guide for genetic mapping in affected individuals. Genetic studies are needed to confirm diagnosis, detect carriers in at-risk family members, and for genetic counseling and prenatal diagnosis.
